# Head-to-head comparisons of the neutralizing antibody against SARS-CoV-2 variants elicited by four priming-boosting regimens

**DOI:** 10.1080/22221751.2022.2095931

**Published:** 2022-07-17

**Authors:** Hu-Dachuan Jiang, Xi-ling Guo, Peng-Fei Jin, Rong Tang, Jing-Xin Li, Feng-Cai Zhu

**Affiliations:** a School of Public Health, Southeast University, Nanjing, People’s Republic of China; b NHC Key laboratory of Enteric Pathogenic Microbiology(Jiangsu Provincial Center for Disease Control and Prevention), Nanjing, People’s Republic of China

To the Editor: Inactivated COVID-19 vaccine (CoronaVac), Ad5-vectored COVID-19 vaccine (Convidecia), and protein subunit COVID-19 vaccine (Zifivax) are widely used in low- and middle-income countries, including China, Brazil, Mexico, Pakistan, Chile, Egypt, Indonesia, Nepal, Turkey, etc. [1]. The emergence of the Delta (B.1.617.2) and Omicron (B.1.1.529) variant of SARS-CoV-2, which coincided with breakthrough cases among vaccinated persons, suggested the need for a third (booster) dose of the SARS-CoV-2 vaccine [2,3]. Several preliminary reports of clinical trials of heterologous or homologous prime-boost immunization regimens with the combination of one or two of these vaccines have been reported [4–6]. Here, we put four heterologous or homologous prime-boost immunization regimens together for head-to-head comparisons of the neutralizing antibody against wild-type SARS-CoV-2, Delta, and Omicron variants.

We have sequentially selected 30 individuals from each of the three heterologous prime-boost immunization cohorts CoronaVac-CoronaVac-Convidecia (intramuscular injection, IM), CoronaVac-CoronaVac-Convidecia (orally inhaled, OI), Convidecia- Zifivax-Zifivax, and from one homologous prime-boost immunization cohort CoronaVac-CoronaVac-CoronaVac. Demographic characteristics and immunization information are presented in Table S1 in the Supplement. Neutralizing antibodies against wild-type H49Y, Delta, and Omicron were measured using a cytopathic effect-based microneutralization assay.

We found that neutralizing antibodies levels against wild-type H49Y (GISAID EPI_ISL_411952) were significantly higher in three heterologous prime-boost immunization groups than that in homologous prime-boost group (Figure 1(A)). CoronaVac-CoronaVac-Convidecia (OI) regimen elicited the strongest neutralizing antibodies against wild-type H49Y, with the geometric mean titers (GMTs) of 675.6 (95% confidence interval [CI], 405.1–1126.6), and 1702.6 (95%CI, 1166.5–2484.3) at day 14 and 28, respectively. Comparable levels of neutralizing antibodies against wild-type H49Y were observed in participants receiving CoronaVac-CoronaVac-Convidecia (IM), and Convidecia-Zifivax-Zifivax, with the GMTs ranged between 161.3 and 181.0 at day 14. While, the lowest GMT of neutralizing antibodies against wild-type H49Y was found in the CoronaVac-CoronaVac-CoronaVac group.

**Figure 1.**
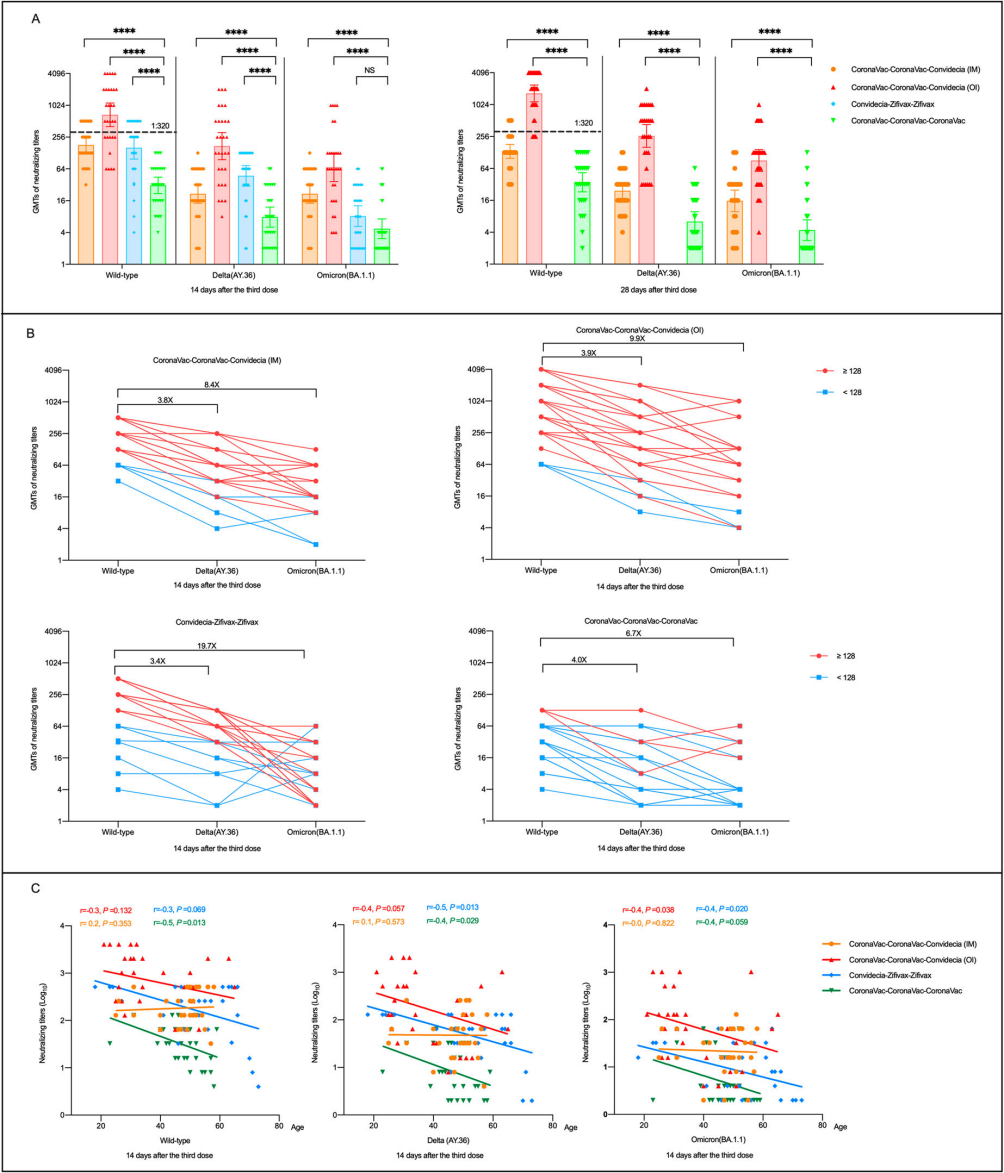
Neutralizing antibodies to wild-type SARS-CoV-2, Delta, and Omicron (BA.1.1) variants after receiving heterologous or homologous prime-boost immmunization regimens. (A) Horizontal dotted line, the WHO reference NIBSC code: 20/136 (1000 IU ml^−1^ in serum) is equivalent to a live viral neutralizing antibody titer of 1:320 against wild-type SARS-CoV-2. Statistical comparisons were performed by using GMTs of heterologous booster groups (CoronaVac-CoronaVac-Convidecia (intramuscular injection, IM), CoronaVac-CoronaVac-Convidecia (orally inhaled, OI), Convidecia-Zifivax-Zifivax) versus that of homologous booster group (CoronaVac-CoronaVac-CoronaVac), respectively; ****P< 0.0001; NS, not statistically. (B) The values are the folds reduction of GMTs of neutralizing antibodies against SARS-CoV-2 wild-type and Delta versus Omicron variants 14 days after the third dose. Red dots represent participants with a neutralizing titer≥1:128 against wild-type SARS-CoV-2. Blue dots represent participants with a neutralizing titer < 1:128 against wild-type SARS-CoV-2. C, Correlation of age and neutralizing antibodies titers to wild-type SARS-CoV-2, Delta, and Omicron variants 14 days after the last boosting dose by four immunization regimens.

Compared to the peak titers against wild-type H49Y, the cross-neutralization of the serum against Delta variant(AY.36)(GISAID EPI_ISL_4515846) were 3.4- to 4.0-folds lower in participants from the four cohorts (Figure 1 (B)). However, the neutralizing antibody responses to Delta variant(AY.36) elicited by heterologous prime-boost immunization were still superior to homologous prime-boost immunization (Figure 1(A)). The highest neutralizing antibodies against Delta variant(AY.36) were observed in the CoronaVac-CoronaVac-Convidecia (OI) group with GMTs of 172.9 (95%CI, 95.7–312.3) at day 14 and 262.0 (95%CI, 160.1–428.7) at day 28, followed by the CoronaVac-CoronaVac-Convidecia (IM) group and the Convidecia-Zifivax-Zifivax group with GMTs of 47.4 (95%CI, 31.9–70.3) and 47.4 (95%CI, 30.2–74.3) at day 14, respectively, and the lowest GMT of 7.8 (95%CI, 5.5–12.1) was found in the CoronaVac-CoronaVac-CoronaVac group. Neutralizing antibodies to Omicron (BA.1.1) variant (EPI_ISL_12511653) decreased more evident with GMTs of 9.9-fold lower in the CoronaVac-CoronaVac-Convidecia (OI) group, 8.4-fold lower in the CoronaVac-CoronaVac-Convidecia (IM) group, 6.7-fold lower in the CoronaVac-CoronaVac-CoronaVac group, and 19.7-fold lower in the Convidecia-Zifivax-Zifivax group than that to wild-type strain (Figure 1(B)). At day 14, the GMTs of neutralizing antibodies against Omicron (BA.1.1) variant were 68.6 (95%CI, 36.8–128.1), 21.6 (95%CI, 14.3–32.6), and 8.2 (95%CI, 5.2–12.8) in the participants received CoronaVac-CoronaVac-Convidecia (OI), CoronaVac-CoronaVac-Convidecia (IM), and Convidecia-Zifivax-Zifivax, respectively, versus 4.7(95%CI, 3.1–7.2) in those received CoronaVac-CoronaVac-CoronaVac (Figure 1(A)). In addition, participants with higher (≥1:128) or lower (<1:128) antibody levels to the wild strain revealed the similar declining trends of the neutralizing antibodies to Delta and Omicron variant.

We found that age was negatively correlated to neutralizing antibodies against all three SARS-CoV-2 strains in heterologous prime-boost groups of CoronaVac-CoronaVac-Convidecia (OI) and Convidecia-Zifivax-Zifivax as well as the homologous prime-boost immunization group of CoronaVac-CoronaVac-CoronaVac, but not in the CoronaVac-CoronaVac-Convidecia (IM) group (Figure 1 (C), and Figure S1). Correlation analyses were not statistically significant for some strains or some groups might be contributed to the small sample size.

**Discussion:** Our data supports that heterologous prime-boost immunization induced significantly higher neutralizing antibodies against wild-type SARS-CoV-2, Delta, and Omicron variants than did homologous three-dose vaccination with CoronaVac. SARS-CoV-2 Delta and Omicron variants, extensively but incompletely escaped humoral immunity elicited by all the four priming-boosting regimens, with various declining magnitudes. SARS-CoV-2 strains used with mutations and different sub lineages might influenced the results of neutralizing responses, so we used WHO international standard (NIBSC code 20/136) for calibration and harmonization. The immune responses post the boosting vaccination decreased with the increasing age was not observed in the CoronaVac-CoronaVac-Convidecia (IM) cohort, which showed a complete different trend to other three regimens. However, the serum were collected from participants in three different prime-boost immunization trials, leading to the slight differences in population characteristics and blood collection time points, which might cause some uncertainty of the results. Another limitation was that the sample size of this study is small, a larger population study is need to further verify our findings.

## Supplementary Material

Supplemental Material
