# Supplementary material for: Head-to-head comparisons of the neutralizing antibody against SARS-CoV-2 variants elicited by four priming-boosting regimens
Source: Emerg Microbes Infect. 2022 Jul 17;11(1):1751–3. doi: 10.1080/22221751.2022.2095931 (PMC9291705; doi:10.1080/22221751.2022.2095931)
Supplement: Supplemental Material [file TEMI_A_2095931_SM9252.doc]

**Supplementary Content**

**Supplementary information, Supplemental Table S1.** Demographic characteristics and immunization information of participants.

**Supplementary information, Figure S1.** The correlation of age and neutralizing antibodies against SARS-CoV-2 variants among participants aged between 18 and 59 years

**Supplemental Table S1. Demographic characteristics and immunization information of participants.**

|  |  | **CoronaVac-CoronaVac-**  **Convidecia (IM)** |  | **CoronaVac-CoronaVac-**  **Convidecia (OI)** |  | **Convidecia-Zifivax**  **-Zifivax** |  | **CoronaVac-CoronaVac-**  **CoronaVac** |
| --- | --- | --- | --- | --- | --- | --- | --- | --- |
| **Age(%)** | | | | | | | | |
| 18-59 |  | 30(100) |  | 28(93.3) |  | 19(63.3) |  | 30(100) |
| ≥60 |  | 0 |  | 2(6.7) |  | 11(36.7) |  | 0 |
| Median age(IQR) |  | 49(45.0-52.3) |  | 32.5(27.0-49.3) |  | 54.0(46.0-63.3) |  | 48(43.8-52.0) |
| **Sex(%)** | | | | | | | | |
| Male |  | 14(46.6) |  | 13(43.3) |  | 15(50.0) |  | 18(60.0) |
| Female |  | 16(53.4) |  | 17(56.7) |  | 15(50.0) |  | 12(40.0) |
| **Prime-boost immunization regimens** | | | | | | | | |
| Prime immunization |  | “0, 28 days” two-dose of CoronaVac |  | “0, 28 days” two-dose of CoronaVac |  | “0 day” one-dose of Convidecia |  | “0, 28 days” two-dose of CoronaVac |
| Boost immunization |  | One dose of Convidecia via intramuscular injection at month 3~6 |  | One dose of Convidecia via orally inhaled at month 3~9 |  | Two doses of Zifivax at day 56 and month 6 |  | One dose CoronaVac at month 3~6 |
| **Time since the last priming dose (months)** | | | | | | | | |
| Median(IQR) |  | 3.9(3.2-4.6) |  | 5.0(3.0-5.0) |  | 2.0(2.0-2.0) |  | 5.0(3.0-5.0) |
| **ClinicalTrials.gov numbers** | | | | | | | | |
|  |  | NCT04892459 |  | NCT05043259 |  | NCT04833101 |  | NCT04892459 |

Data are n(%) or median (IQR);

IM, intramuscular injection; OI, orally inhaled;

**Figure S1. The correlation of age and neutralizing antibodies against SARS-CoV-2 variants among participants aged between 18 and 59 years***


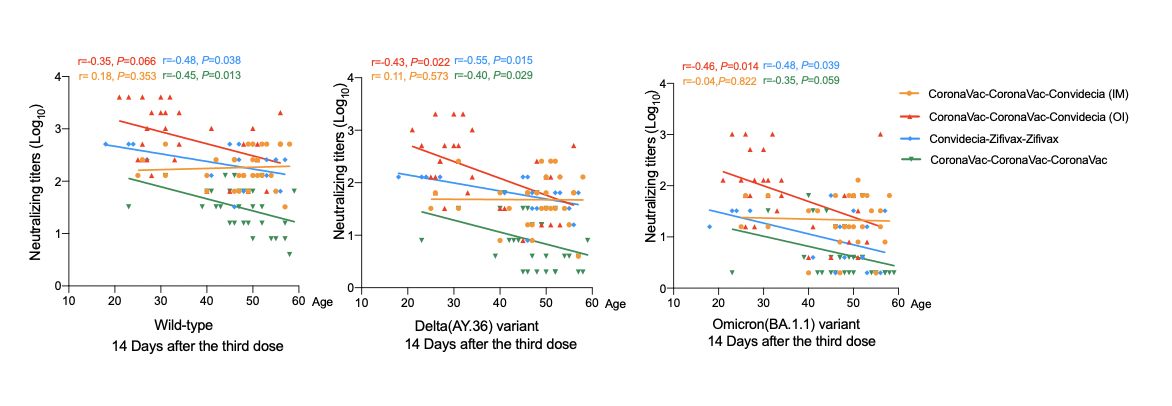


*Two participants aged≥60 years were excluded from CoronaVac-CoronaVac-Convidecia (OI) group and 11 participants aged≥60 years were excluded from Convidecia-Zifivax-Zifivax group.

IM, intramuscular injection; OI, orally inhaled.
